# Deficiency of the histone H3K36 methyltransferase SETD2 inhibits the proliferation and migration of hepatocellular carcinoma cells

**DOI:** 10.7150/jca.97844

**Published:** 2024-10-21

**Authors:** Yi Yang, Linlin Zhang, Gustave Munyurangabo, Ying Zhou, Shuyang He, Peihua Zhang, Xiao Yu, Guangyao Kong

**Affiliations:** 1 Department of Oncology, National-Local Joint Engineering Research Center of Biodiagnostics and Biotherapy, The Second Affiliated Hospital of Xi'an Jiaotong University, Xi'an, Shaanxi, People's Republic of China.; 2 Center for Tumor and Immunology, the Precision Medical Institute, Xi'an Jiaotong University, Xi'an, Shaanxi, People's Republic of China.; 3 Queen Mary school, Nanchang University, Nanchang, Jiangxi, People's Republic of China.

**Keywords:** SETD2, hepatocellular carcinoma, FGFBP1, H3K36me3

## Abstract

**Background:** Hepatocellular carcinoma (HCC) is one of the leading causes of cancer-related death worldwide. SETD2, the only known methyltransferase catalyzes the trimethylation of histone H3 lysine 36 (H3K36), has been reported to be associated with several cancers. However, the function of SETD2 in HCC is unclear. This work aimed to investigate the function and mechanism of SETD2 in HCC through bioinformatics analysis and cell experiments.

**Methods:** SETD2 expression and its relationship with prognosis were evaluated in The Cancer Genome Atlas (TCGA)-LIHC cohort, and the effects of SETD2 silencing and overexpression on HCC cell lines were assesed via CCK-8, colony formation and wound healing assays. RNA-seq analysis, western blotting and chromatin immunoprecipitation (ChIP) assays were used to assess the potential mechanism of action of SETD2 in HCC.

**Results:** The results indicated that SETD2 expression is upregulated and that high SETD2 expression is related to a poor prognosis in HCC patients. SETD2 silencing inhibited proliferation and migration, and SETD2 overexpression promoted proliferation and migration in HCC cells. RNA-seq data revealed that the differentially expressed genes were enriched in the fibroblast growth factor receptor signaling pathway. FGFBP1, which was an FGF-binding protein and could enhance FGFR signaling pathway by releasing FGF from the extracellular matrix, was among the top 10 DEGs. Furthermore, the expression of FGFBP1 was decreased in SETD2-silenced BEL-7402 cells. The expression level of phosphorylated ERK, a downstream effector of FGFR, was positively correlated with the expression level of SETD2. In addition, ChIP-qPCR confirmed that the H3K36me3 modification occured on the gene body of FGFBP1.

**Conclusions:** Our findings highlight the role of SETD2/H3K36me3 in promoting HCC proliferation and migration via the FGFR pathway. Our study advances our understanding of epigenetic dysregulation during HCC progression and provides a rationale for the application of SETD2 as a potential diagnostic biomarker and therapeutic target in HCC.

## Introduction

Primary liver cancer is one of the major causes of cancer-related death worldwide among all primary liver cancers, hepatocellular carcinoma (HCC) is the most common neoplasm 1. The global burden of HCC is steadily growing, and various risk factors for HCC development, such as chronic hepatitis B virus (HBV) and hepatitis C virus (HCV) infections, cirrhosis (chronic liver damage caused by inflammation and fibrosis), aflatoxin B1 intake produced by A. flavis and A. parasitan, chronic alcohol abuse and metabolic syndrome, obesity and diabetes mellitus, etc., have been defined 1,2. Despite advances in HCC therapy involving chemoradiotherapy, surgery and targeted therapy, the mortality rate of HCC is still high due to the lack of effective diagnostic methods and therapeutic schedule 2-4. Therefore, studies exploring the carcinogenic mechanism and screening the targets of diagnosis and therapy are urgently needed.

Epigenetics is the study of heritable changes in nuclear chromatin that occur via modification rather than changes in nucleotide sequence; these modifications include noncoding RNA methylation, DNA methylation and histone modification 5. Growing evidence indicates that aberrant epigenetic regulation has an important role in the development and progression of various tumors, including hepatocellular carcinoma 6,7. Histone modification is a reversible epigenetic process, and new anticancer drugs related to lysine methyltransferases and demethylase targets have been explored 8,9. Set domain-containing 2 (SETD2, also known as Huntingtin interacting protein B [HYPB]), a histone methyltransferase, can catalyze the trimethylation of histone H3 lysine 36 (H3K36) and participate in a variety of biological processes, including transcriptional elongation, alternative splicing and DNA repair 10. In addition, SETD2 mutation is associated with the development and progression of several cancers, such as clear cell renal cell carcinoma, breast carcinoma and colorectal cancer, and is involved in chemotherapy resistance in leukemia patients 7,10-12. Previous studies have shown that SETD2 and H3K36me3 are closely correlated with the development and progression of hepatocellular carcinoma, but there are different ideas about whether SETD2 expression and H3K36me3 promote or inhibit hepatocellular carcinoma progression 13-15. Furthermore, the underlying mechanism of SETD2 in the development and progression of hepatocellular carcinoma is largely unknown.

Fibroblast growth factor-binding protein 1 (FGFBP1) is a secreted protein that binds to FGF1, 2, 7, 10 and 22 in a reversible manner via its C-terminal domain, which enhances FGF functions by releasing FGFs from the extracellular matrix and binding to their cognate FGF receptors 16. The FGF/FGFR family, which consists of 4 receptors and 19 ligands, is highly important in several human cancers and is involved in the occurrence, angiogenesis and development of HCC 17,18. The interaction of FGFs with their receptors leads to the activation of downstream pathways such as the ERK signaling pathway 19,20. The FGFR pathway plays an important role in a number of cellular processes, including proliferation, metabolism, differentiation and migration, and is involved in the progression of cancers such as breast cancer, esophageal squamous cell carcinoma, and colon cancer 20-22. FGFBP1 is related to embryonic development, wound healing, and angiogenesis, and its expression is low in adult human tissues. Overexpression of FGFBP1 promotes colon carcinoma, pancreatic cancer, breast cancer and skin carcinogenesis 23,24. In addition, sox12 promotes hepatocellular carcinoma through upregulating twist1 and FGFBP1 16. These findings suggest the important role of FGFBP1 in cancer occurrence and development.

Here, we evaluated the mRNA expression and prognostic value of SETD2 in LIHC patients by analyzing data from The Cancer Genome Atlas (TCGA) database. Furthermore, the function of SETD2 in HCC cell lines was explored. Additionally, we investigated the mechanism of action of SETD2 in HCC cell lines.

## Materials and Methods

### Data acquisition and bioinformatics analysis

The datasets used in this study were downloaded from the TCGA database (https://portal.gdc.cancer.gov/). RNA-seq and corresponding patient data were obtained for 374 liver hepatocellular carcinoma (LIHC) samples and 50 normal tissue samples. Pancancer RNA-seq data were obtained for 10363 cancer samples and 730 normal tissue samples. The RNA-seq data and clinical patient data (HTseq-FPKM) were obtained from the Data Transfer Tool (provided by GDC Apps). The level 3 HTSeq-FPKM data were log2 transformed for the subsequent analyses. LIHC patients were classified into low- and high-expression groups according to the minimum P value in prognostic analysis. This study used R (version 3.6.3) to analyze and visualize SETD2 mRNA expression and clinical data. Survival data were obtained from a published paper (https://www.sciencedirect.com/science/article/pii/S0092867418302290?via%3Dihub) for external validation. The paired-sample t test and Wilcoxon rank-sum test were used to analyze the expression of SETD2 in paired and nonpaired LIHC patients, respectively, with the ggplot2 package. The ROC curve was used to evaluate the diagnostic performance of SETD2 mRNA expression with the pROC and ggplot2 packages. The Kaplan‒Meier method was used to construct survival curves, and the differences between two groups were evaluated via the log-rank test with the survminer and survival packages.

### Human clinical samples

HCC tissues and matched adjacent nontumorous liver tissues were obtained from patients who underwent surgical treatment at the Second Affiliated Hospital of Xi'an Jiaotong University. All samples were pathologically confirmed before collection.

### Cell culture

The human HCC cell lines Bel-7402, SMMC-7721, HepG2, Hep3B, MHCC97-L, and SK-Hep1 and the normal human liver cell line L02 were obtained from the Cell Bank of Type Culture Collection of the Chinese Academy of Sciences (Shanghai, China). All cell lines were cultured in Dulbeccoʼs modified Eagleʼs medium (BasalMedia) supplemented with 10% fetal bovine serum (FBS, ExCell).

### Vector construction, RNA interference and transfection

For SETD2 silencing, we used small interfering RNAs (siRNAs) synthesized by RiboBio (China). The siRNA sequences used were as follows: si-SETD2-02, 5'-GGAGTATGCACGAAACAAA-3'; si-SETD2-03, 5'-GCTCAGAGTTAACGTTTGA-3'. The siRNAs were subsequently transfected into cells using Lipofectamine™ RNAiMAX (Invitrogen, USA) at a final concentration of 20 nM according to the manufacturer's protocol. For SETD2 overexpression, the open reading frame (ORF) of human SETD2 (2857-5202 bp) was amplified and inserted into the mammalian expression vector pcDNA3.1 (Clontech, USA), and the resulting plasmid (SETD2-F2) was transfected using Lipofectamine® 3000 Reagent (Invitrogen, USA) according to the manufacturer's protocol.

### RNA extraction and RT-PCR

Total RNA was extracted from tissues or cells using RNAiso Plus reagent (Takara), first-strand complementary DNA was prepared with PrimeScript RT Master Mix (Takara), and quantitative real-time polymerase chain reaction (RT-PCR) was performed using TB Green Premix Ex Taq II (Takara) with a 7500 Fast Real-Time PCR System (Applied Biosystems); β-actin was used as the endogenous control.

### Western blotting

Total protein was extracted from the cells with M-PER Mammalian Protein Extraction Reagent (Thermo Fisher Scientific), and the protein concentration was subsequently quantified via a BCA protein assay kit (TianGen). Fifty micrograms of protein lysate were separated by 10% sodium dodecyl sulfate‒polyacrylamide gel electrophoresis and then transferred electrophoretically onto a polyvinylidene difluoride membrane (0.22 μm, GE Healthcare). Then, the membrane was incubated with primary antibodies and horseradish peroxidase-conjugated secondary antibodies in TBST. The chemiluminescence method was used to detect the protein bands using Immobilon Western Chemiluminescent HRP Substrate (Millipore). The primary antibodies used in this study were as follows: GAPDH (14C10), a rabbit monoclonal antibody (mAb) (Cell Signaling Technology, 2118); and SETD2 (E4W8Q), a rabbit mAb (Cell Signaling Technology, 80290). Phospho-p44/42 MAPK (Erk1/2) (Thr202/Tyr204) (D13.14.4E) XP® Rabbit mAb (Cell Signaling Technology, 4370).

### CCK-8 assay

Cell viability was assessed with a Cell Counting Kit-8 (UElandy, C6005) assay according to the manufacturer's protocol. Cells were seeded at a density of 2600 cells/well in 96-well plates and incubated for a certain time. Then, 10 µl of Cell Counting Kit-8 reagent was added to each well, and the cells were incubated for 2 hours at 37 °C in a humidified incubator. The optical density at 450 nm was measured by a microplate reader.

### Colony formation assay

Cells were seeded at a density of 500 cells/well in 6-well plates and cultured for 14 days. Colonies were stained with crystal violet staining solution (Solarbio, G1075) for 15 minutes, and colonies containing ≥20 cells were counted.

### Wound healing assay

The cells were seeded in 6-well plates and cultured until they reached 100% confluence. A pipette tip was subsequently used to make straight scratches. The cells were observed using a microscope (×10) at the indicated times.

### RNA-sequencing analysis

The cells were transfected with si-SETD2-03 or si-NC, and we collected and mixed the cells from three experiments. Total RNA was subsequently extracted from these cells using RNAiso Plus reagent (Takara), after which the RNA quality was measured via an Agilent 2100 Bioanalyzer (Agilent Technologies). cDNA library construction and sequencing were performed by chi Biotech using an Illumina HiSeq™ 2000. The fragments per kilobase of transcript per million mapped reads (FPKM) were used to evaluate the gene expression levels via expectation maximization via the RSEM package.

### ChIP-qPCR

ChIP assays were performed using the Piece Agarose ChIP Kit (Thermo Scientific) according to the manufacturer's instructions. A total of 2 × 106 cells was cross-linked with 1% paraformaldehyde and then fragmented with MNase. A ChIP-grade anti-H3K36me3 antibody (Cell Signaling Technology, 4909) was used for immunoprecipitation. After elution and DNA recovery, the purified DNA was amplified using CCDC82 primers and then quantified using TB Green Premix Ex Taq II (Takara) with a 7500 Fast Real-Time PCR System (Applied Biosystems). The data were normalized as a percentage of the input.

### Statistical analysis

All the statistical analyses were performed using SPSS 20.0. The data are expressed as the means ± standard deviations (SD). Differences between two groups and multiple groups were determined by Student's t test and one-way analysis of variance (ANOVA), respectively. The expression of SETD2 in unpaired or paired HCC tissues was analyzed by the Mann-Whitney U test or Wilcoxon signed rank test, respectively. p<0.05 was considered to indicate statistical significance.

## Results

### SETD2 is upregulated, and its high expression is related to a poor prognosis in HCC patients

**Figure 1:**
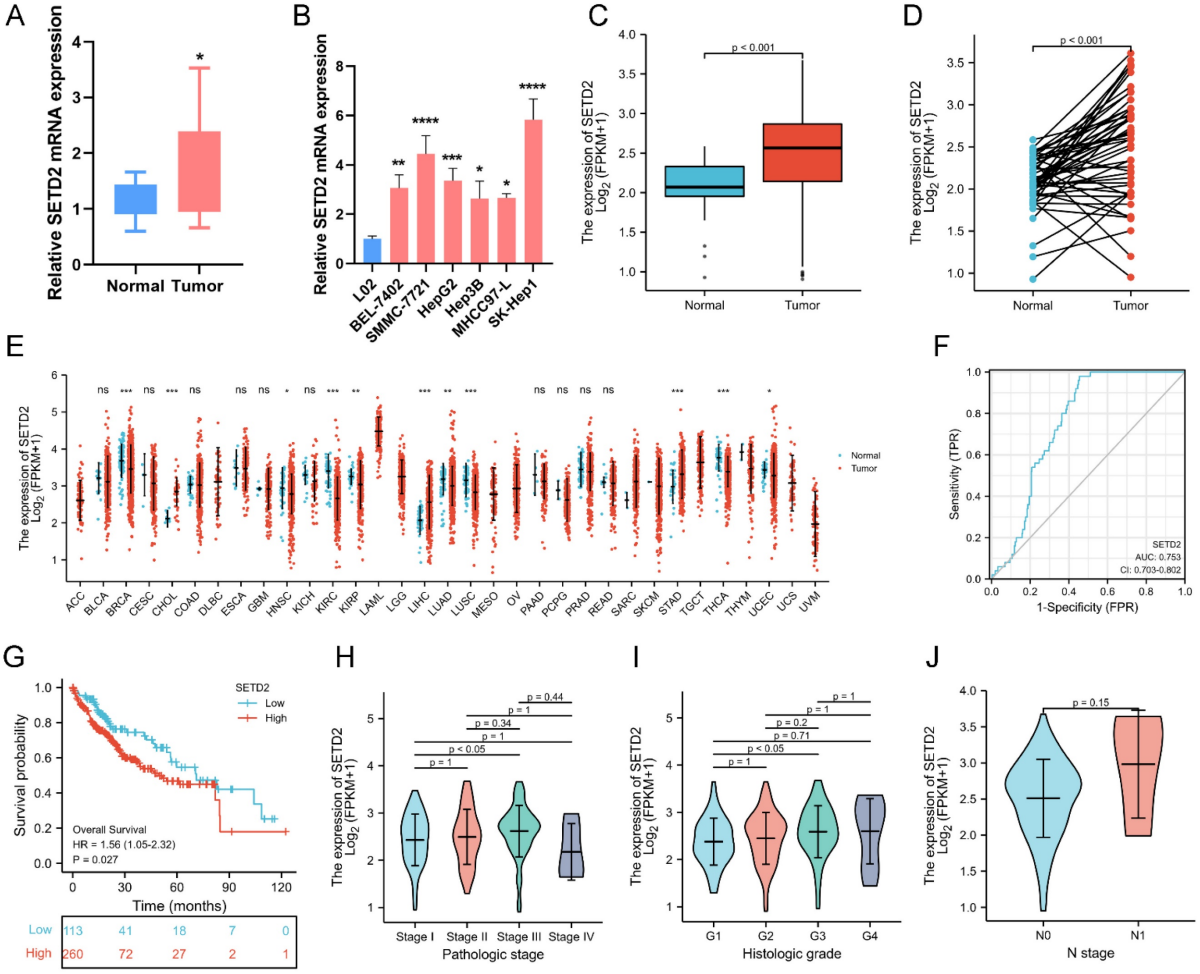
SETD2 expression is increased, and its high expression is related to a poor prognosis in HCC patients. **A** Expression levels of SETD2 in human HCC tissues (tumor) and impaired adjacent peritumoral tissues (normal) measured by qRT‒PCR; **B** Expression levels of SETD2 in 6 HCC cell lines and a normal liver cell line (L02) measured via qRT‒PCR. **C** Expression levels of SETD2 in normal tissue (n=50) and LIHC tissue (n=374) from the TCGA-LIHC database. **D** Expression levels of SETD2 in LIHC (n=50) and paired adjacent peritumoral tissues (n=50) from the TCGA-LIHC database. **E** Expression levels of SETD2 in different tumor types from the TCGA-ALL database. **F** Receiver operating characteristic analysis (ROC) of SETD2 expression in LIHC patients (n=374) from the TCGA-LIHC database. **G** Survival curves of LIHC patients from the TCGA-LIHC database. The LIHC patients were grouped into low expression of SETD2 (n=113) and high expression of SETD2 (n=260). **H** The correlation between SETD2 expression and pathological stage in LIHC patients; **I** The correlation between SETD2 expression and histological grade in LIHC patients; **J** The correlation between SETD2 expression and N stage in LIHC patients. The data are presented as the means ± SDs (n = 3; *P <0.05, **P < 0.01, ***P < 0.005, and ****P < 0.001).

To investigate the role of SETD2 in human HCC, we first evaluated the expression of SETD2 in HCC patients and HCC cell lines by qRT‒PCR. The results showed that SETD2 expression was upregulated in human HCC tumor tissues (Figure 1A) and in 6 HCC cell lines (Figure 1B) compared with that in impaired adjacent peritumoral tissues and normal liver cell lines, respectively. Subsequently, we assessed the expression of SETD2 in LIHC patients via the TCGA database. The data indicated that SETD2 was upregulated in LIHC tumor tissue compared with normal tissue (Figure 1C, P < 0.001). In paired samples, the expression of SETD2 in the LIHC group was greater than that in the paired adjacent peritumoral tissues (Figure 1D, P < 0.001). SETD2 expression in various human cancers was also examined using pancancer RNA-seq data from The Cancer Genome Atlas (TCGA) database. The results (Figure 1E) showed that SETD2 expression was upregulated in tumor vs. normal tissues in the cholangiocarcinoma (CHOL), hepatocellular carcinoma (LIHC) and stomach adenocarcinoma (STAD) and that SETD2 expression was downregulated in invasive breast carcinoma (BRCA), head-neck squamous cell carcinoma (HNSC), kidney renal clear cell carcinoma (KIRC), kidney renal papillary cell carcinoma (KIRP), lung adenocarcinoma (LUAD), lung squamous cell carcinoma (LUSC), thyroid carcinoma (THCA) and uterine corpus endometrial carcinoma (UCEC) datasets. The expression levels of SETD2 in most tumors vs. normal tissues were downregulated, and SETD2 inhibits the growth of these tumor, for example, in lung adenocarcinoma 7 and in kidney cancer 11. The pancancer RNA-seq data from TCGA database showed that the expression of SETD2 was upregulated in hepatocellular carcinoma, so SETD2 might play oncogene role in hepatocellular carcinoma.

The diagnostic value of SETD2 in patients with LIHC was assessed using receiver operating characteristic (ROC) analysis, and the area under the curve (AUC) was 0.753 (95% CI=0.703-0.802) (Figure 1F). We also analyzed the survival data of 373 LIHC patients from the TCGA-LIHC cohort to assess the relationship between SETD2 expression and the prognosis of LIHC patients. The LIHC samples were divided into two groups according to the expression level of SETD2 (low or high). Kaplan-Meier survival curves were generated, and the analysis showed that patients with high SETD2 expression had worse overall survival (OS) than patients with low SETD2 expression (HR=1.56, 95% CI=1.05-2.32; p=0.027; Figure 1G). Moreover, the correlations between SETD2 expression and clinical characteristics were explored, and the results revealed that SETD2 expression was associated with pathological stage, histological grade, N stage and residual tumor status (Figure 1H-I); other clinical characteristics were not correlated with SETD2 expression, and detailed information about these associations is not provided in this paper. These data showed that the high expression of SETD2 predicts a poor prognosis and SETD2 might play oncogene role in hepatocellular carcinoma. Lien HC et al reported that increased trimethylation of histone H3K36 predicted poor prognosis in hepatocellular carcinoma 15, this finding was consistent with our conclusion.

In summary, the above findings revealed that SETD2 is upregulated in HCC patients and that its high expression predicts a poor prognosis.

### SETD2 silencing inhibits the proliferation and migration of HCC cell lines

**Figure 2:**
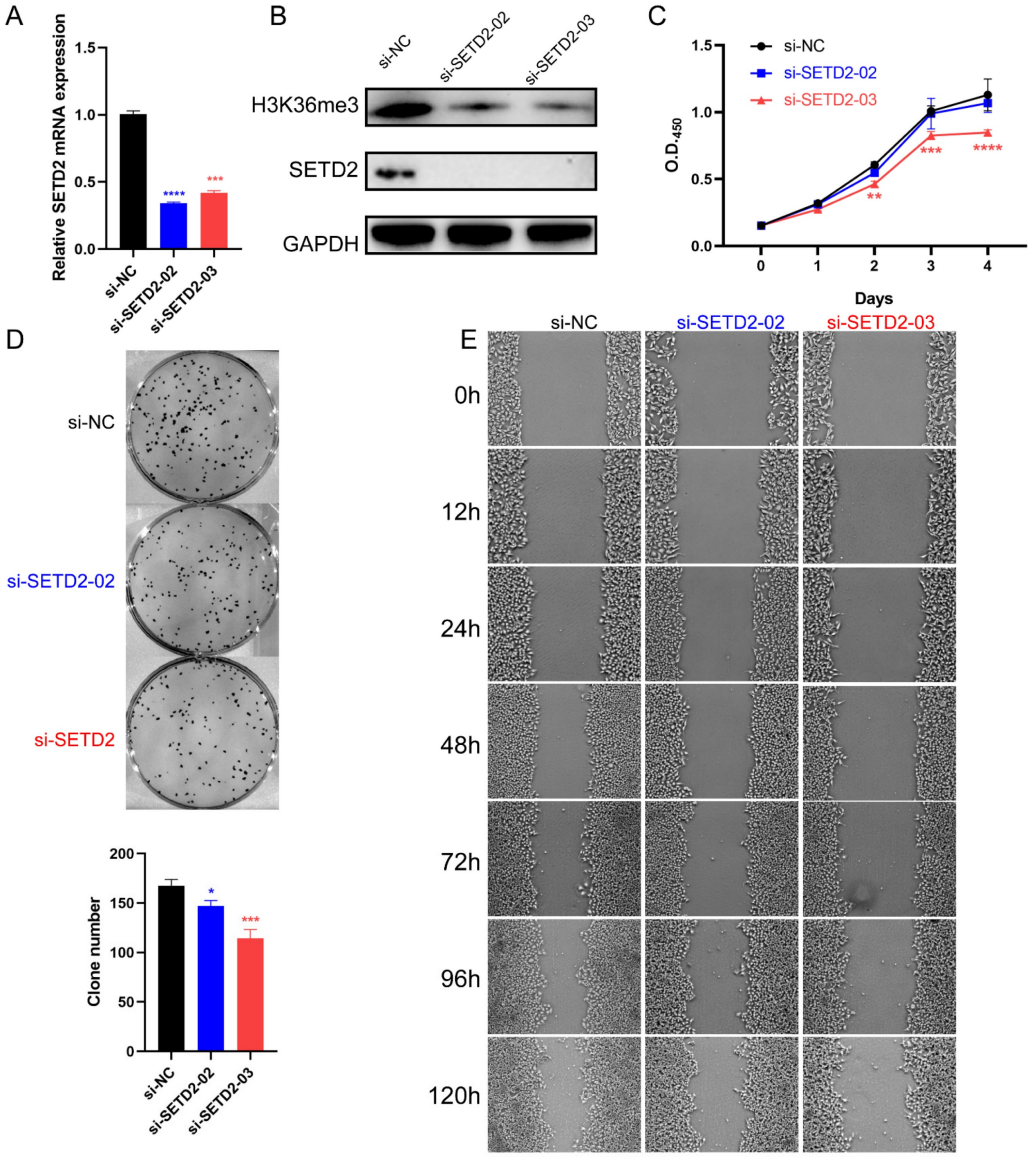
SETD2 silencing inhibited the proliferation and migration of HCC cell lines. **A** The efficiency of SETD2 silencing in BEL-7402 cell lines was measured by qRT‒PCR. **B** The efficiency of SETD2 silencing in BEL-7402 cell lines was examined by western blotting. **C** The efficiency of SETD2 silencing on the proliferation of HCC cell lines was determined via CCK-8 assay. **D** The efficiency of SETD2 silencing on the proliferation of HCC cell lines was evaluated via colony formation assays. **E** The efficiency of SETD2 silencing on the migration of HCC cell lines was detected via a scratch wound healing (cell migration) assay, the pictures were screenshots from the same position of origin picture as shown in supplement material (Figure S6) using Photoshop (data are given as the mean ± SD, n = 3; *P <0.05, **P < 0.01, ***P < 0.005, and ****P < 0.001).

To determine whether SETD2 affects the proliferation and migration of HCC cell lines, we performed a loss-of-function experiment in BEL-7402 cells. The silencing efficiency of si-SETD2 was evaluated by qRT‒PCR and western blotting. The results showed that SETD2 mRNA expression was downregulated after 24 hours in cells transfected with 20 nM si-SETD2-02 or si-SETD2-03 compared with cells transfected with si-NC (Figure 2A). The protein levels of SETD2 and its relevant modification H3K36me3 were decreased after 48 hours in cells transfected with 20 nM si-SETD2-02 or si-SETD2-03 compared with those transfected with si-NC (Figure 2B). These data indicated the ability of si-SETD2 to silence SETD2. Subsequently, we assessed the impact of SETD2 silencing on the proliferation of HCC cell lines via CCK-8 assays, and the results showed that the proliferation ability of BEL-7402 cells was decreased upon si-SETD2-03 transfection but not upon si-SETD2-02 transfection (Figure 2C). Additionally, a colony formation assay indicated that si-SETD2 decreased the colony formation ability of the BEL-7402 cell line (Figure 2D). Furthermore, a scratch wound healing (cell migration) assay was used to evaluate the migration of the HCC cell line BEL-7402. We marked the wound margins with a red line for visualization. The images indicated that SETD2 silencing, especially with si-SETD2-03, could inhibit the migration of BEL-7402 cells (Figure 2E). These findings suggested that SETD2 silencing inhibits the proliferation and migration of HCC cell lines which was consistent with our assumption as mentioned above.

### SETD2 overexpression promotes the proliferation and migration of HCC cell lines

**Figure 3:**
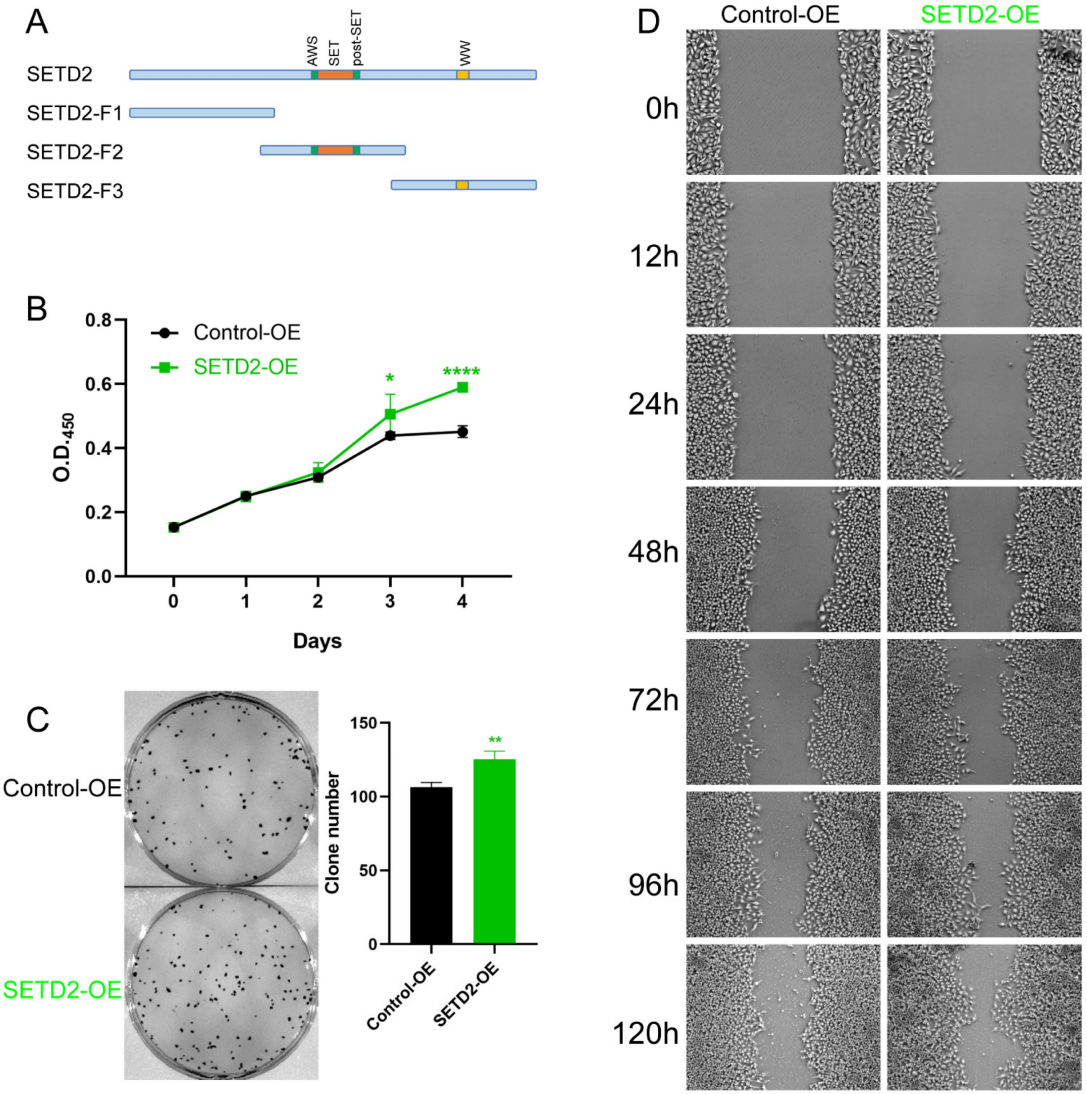
SETD2 overexpression promoted the proliferation and migration of HCC cell lines. **A.** Schematic diagram showing the major domains of SETD2 and the truncated fragments. Fragment 2 (F2) was used in the subsequent experiments. **B**. The effect of SETD2 overexpression on the proliferation of HCC cell lines was determined via a CCK-8 assay. **C.** The effect of SETD2 overexpression on the proliferation of HCC cell lines was evaluated by colony formation assays. **D.** The effect of SETD2 overexpression on the migration of HCC cell lines was detected by a scratch wound healing (cell migration) assay, the pictures were screenshots from the same position of origin picture as shown in supplement material (Figure S7) using Photoshop (data are given as the mean ± SD, n = 3, *P <0.05, **P < 0.01, ***P < 0.005, and ****P < 0.001).

To further investigate whether SETD2 affects the proliferation and migration of HCC cell lines, we constructed vectors encoding amino acids 1-952 (aa), 953-1734 aa (containing the SET domain) and 1735-2565 aa of SETD2 and defined them as SETD2-F1, SETD2-F2, and SETD2-F3, respectively (Figure 3A). Pretest experiments showed that SETD2-F1 and SETD2-F3 had no effect on the proliferation of HCC cell lines; therefore, we used SETD2-F2 (defined as SETD2 overexpression) in the following work. CCK-8 assays indicated that SETD2 overexpression promoted the proliferation of the HCC cell line BEL-7402 (Figure 3B). A colony formation assay showed that SETD2 overexpression increased the colony formation ability of the BEL-7402 cell line (Figure 3C), and the migration ability of the BEL-7402 cells was also enhanced according to the scratch wound healing (cell migration) assay (Figure 3D). The results from SETD2 silencing and SETD2 overexpression experiments demonstrated that SETD2 could promote the proliferation and migration of HCC cell lines; that is, SETD2 has an important role in promoting hepatocellular carcinoma.

### SETD2 silencing inhibits the progression of HCC through FGFR-ERK pathway

**Figure 4:**
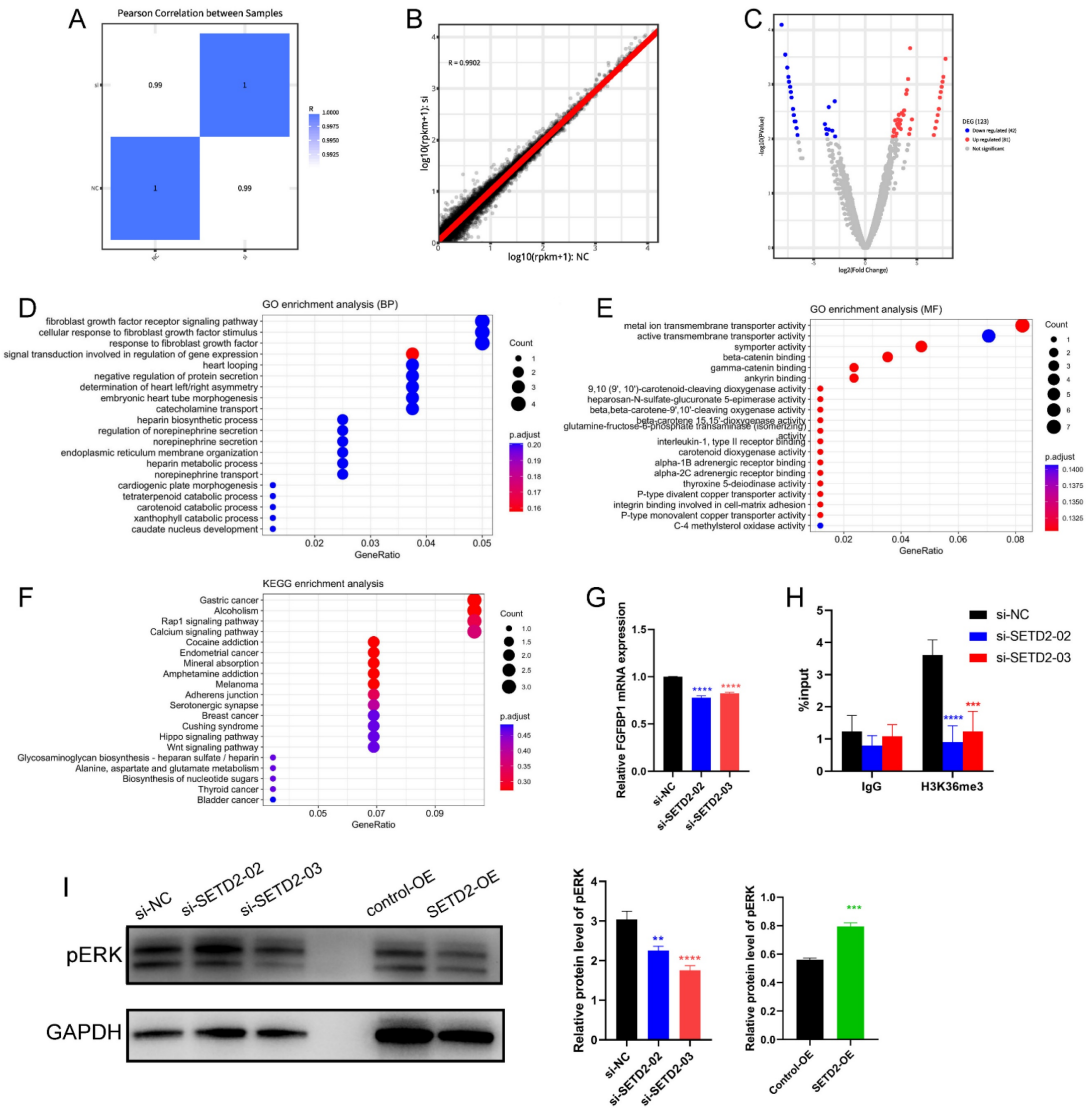
SETD2 silencing inhibited HCC progression by negatively regulating the FGFR-ERK pathway. **A** Pearson correlation with heatmap between in SETD2-silenced (si-SETD2-03) and negative control (si-NC) BEL-7402 cells; **B** Pearson correlation with scatter diagram between in si-SETD2-03 and si-NC cells. **C** Volcano plots displaying a total of 123 DEGs between SETD2 silencing and negative control BEL-7402 cells, the, the log2 (Fold Change) is plotted on X axis and -log10 (p value) is plotted on Y axis, red and blue point represents upregulated and downregulated gene with p value < 0.01 and |log2(FC)| ≥1, respectively. **D** GO enrichment analysis of downregulated DEGs (biological process). **E** GO enrichment of DEGs (molecular function); **F** KEGG pathway enrichment of DEGs. **G** Verification of RNA-seq results through qRT‒PCR. **H** ChIP‒qPCR assays of H3K36me3 in the FGFBP1 gene body in normal and SETD2 silencing NB4 cells.** I** Western blot of pERK expression in control, SETD2-silenced and SETD2-overexpressing BEL-7402 cells (data are given as the mean ± SD, n = 3, *P <0.05, **P < 0.01, ***P < 0.005, and ****P < 0.001).

To explore the potential SETD2-controlled genes in HCC cell lines in more depth, we identified genes that were differentially expressed in SETD2-silenced (si-SETD2-03) and negative control (si-NC) BEL-7402 cells according to RNA-seq. The Pearson correlation with heatmap (Figure 4A) and scatter diagram (Figure 4B) between SETD2-silenced cells and negative control BEL-7402 cells indicated good biological replicates, and we identified 123 DEGs (p value < 0.01 and log2(FC)| ≥1), 42 of which were downregulated and 81 of which were upregulated in SETD2-silenced cells (Figure 4C). Subsequently, we executed GO (Figure 4D and 4E) and KEGG (Figure 4F) analyses of the DEGs via the DAVID tool to identify enriched signaling pathways and enriched biological processes.

The GO results indicated that the DEGs (Figure 4D) were enriched in the fibroblast growth factor receptor (FGFR) signaling pathway, cellular response to fibroblast growth factor stimulus, response to fibroblast growth factor, etc. in the biological process category and that the DEGs (Figure 4E) were enriched in metal ion transmembrane transporter activity, active transporter activity, symporter activity, etc. in the molecular function category. The KEGG analysis (Figure 4F) indicated that DEGs were enriched in several cancer-associated signaling pathways, including the Wnt, Hippo and Rap1 signaling pathways; alcoholism; and the calcium signaling pathway. These data indicated that SETD2 silencing could influence several cancer-associated signaling pathways and affect the fibroblast growth factor receptor signaling pathway. The DEGs enriched in the cellular response to fibroblast growth factor stimulus and fibroblast growth factor receptor signaling pathway were FGF12, FGF5, FGFBP1 and SPRY1; we selected FGFBP1, which ranked among the top 10 downregulated DEGs, for further study. As a potential SETD2-regulated gene, the expression of FGFBP1 (Figure 4G) was downregulated in SETD2-silenced BEL-7402 cells. Moreover, we used ChIP‒qPCR to determine how SETD2-mediated H3K36me3 regulates FGFBP1 expression. The results (Figure 4H) showed that the genomic region of the FGFBP1 gene had a H3K36me3 modification, SETD2 deficiency led to a decrease in the H3K36me3 level, and the H3K36me3 modification could occur directly on the gene body of FGFBP1. FGFBP1 could enhance FGFR signaling pathway by releasing FGF5 and FGF12 from the extracellular matrix which were verified by the RNAseq data 25-27, ERK was the main downstream effector of FGFR signaling pathway, we proposed that SETD2 promoted proliferation and migration of HCC cells through ERK, so we also assessed the ERK phosphorylation level of HCC cells by western blotting; the results (Figure 4I) showed that SETD2 overexpression could enhance ERK phosphorylation level and SETD2 silencing could decrease ERK phosphorylation level in BEL-7402 cells. These findings indicated that SETD2 deficiency inhibits the proliferation and migration of HCC cells through decreasing the H3K36me3 modification level on the FGFBP1 gene body through the FGF-FGFR-ERK axis.

## Discussion

As a histone methyltransferase, SETD2 can catalyze the trimethylation of histone H3 lysine 36 (H3K36) and participate in some biological processes, such as transcriptional elongation, alternative splicing, DNA repair and so on 10. Besides, SETD2 is associated with development and progression of several cancers, such as clear cell renal cell carcinoma, breast carcinoma and colorectal cancer, and is involved in chemotherapy resistance in some patients with tumor 7,10-12. SETD2 and H3K36me3 are closely correlated with the development and progression of hepatocellular carcinoma, but there are different ideas about whether SETD2 expression and H3K36me3 promote or inhibit hepatocellular carcinoma progression 13-15.

Although the SETD2 mutation rate in HCC patients is only ~5%, SETD2 mutations in the liver are associated with the occurrence of spontaneous HCC and the progression of HCC induced by DEN and a high-fat diet *in vivo*; animal experiments revealed that SETD2 represses HCC tumorigenesis by maintaining lipid homeostasis and that SETD2 deficiency causes abnormal expression of cholesterol metabolism genes and lipid accumulation; these increases in lipid levels activate c-Jun, inhibit p53 activation and induce HCC 14. However, the bioinformatic analysis of 374 LIHC patients from the TCGA database (Figure 1) indicated that SETD2 was upregulated in HCC and that high SETD2 expression was related to a poor prognosis. Additionally, the expression of SETD2 in HCC patients and HCC cell lines (Figure 1) was confirmed to be upregulated. In addition, increased levels of SETD2-catalyzed H3K36me3 modification predicted a poor prognosis in patients with hepatocellular carcinoma 16. These studies indicated that SETD2 may promote the progression of HCC, and SETD2 may play a positive role in HCC patients and HCC cell lines. As an epigenetic regulation factor, SETD2 has made complex effect in occurrence and progression of HCC.

Subsequently, the functions of SETD2 in the HCC cell line BEL-7402 were investigated, and we found that SETD2 silencing inhibited the proliferation and migration of BEL-7402 cells. SETD2 overexpression promoted the proliferation and migration of BEL-7402 cells, which indicated that SETD2 has a major role in HCC progression. Finally, we studied the mechanism of action of SETD2 in HCC, and the RNA-seq results (Figure 4E) showed that SETD2 silencing inhibited the proliferation and migration of HCC cell lines through the fibroblast growth factor receptor (FGFR) signaling pathway. As a chaperone protein, FGFBP1 binds to FGF via its C-terminal domain, which enhances FGF functions by releasing FGFs from the extracellular matrix and binding to their cognate FGF receptors, thereby enhancing the activation of FGF/FGFR signaling pathway 16,25,26. The interaction of FGFs with their receptors leads to the activation of downstream pathways such as the ERK signaling pathway 19,20. The FGFR pathway plays an important role in a number of cellular processes, including proliferation, metabolism, differentiation and migration, and is involved in the progression of cancers such as breast cancer, esophageal squamous cell carcinoma, and colon cancer 20-22. Abnormal FGF/FGFR signaling is related to the occurrence of breast cancer, gastric cancer, endometrial cancer, bladder cancer, myeloma, and HCC 17,18. Bevacizumab-resistant tumor cell-derived FGFBP1 induced fibroblast activation protein α expression by enhancing the paracrine FGF2/FGFR1/ERK1/-2/EGR1 signaling pathway in hepatic stellate cells 27. Therefore, the level of phosphorylated ERK in BEL-7402 cells was also investigated. Western blot analysis (Figure 4I) showed that SETD2 silencing decreased the level of phosphorylated ERK and that SETD2 overexpression increased the level of phosphorylated ERK in HCC cells. SETD2 promotes the transcription of FGFBP1 by changing the H3K36me3 level of the FGFBP1 promoter according to the UCSC Genome Browser database, and the direct binding of H3K36me3 to the FGFBP1 gene body was confirmed via ChIP-PCR (Figure 4H). These findings emphasized that SETD2 deficiency inhibits the proliferation and migration of HCC cells through decreasing the H3K36me3 level on the FGFBP1 gene body through the FGF-FGFR-ERK axis. Although our finding demonstrated that SETD2 promoted the progression of HCC *in vitro*, the function of SETD2 *in vivo* was still unclear and we should research the function of SETD2 in HCC *in vivo* in the future.

In conclusion, our finding reveals the molecular mechanism of SETD2 in HCC *in vitro*, in which SETD2 may mediate the proliferation and migration of HCC cells through FGFBP1-FGF-FGFR-ERK axis.

## Conclusion

In this work, we evaluated the function and mechanism of SETD2 in HCC through bioinformatics analysis and cell experiments, and our findings highlighted the promoting role of SETD2 in HCC cell proliferation and migration, which will guide further studies about the role of SETD2 and FGFBP1 in HCC tumorigenesis and progression.

## Supplementary Material

Supplementary figures.
