# Supplementary material for: Deficiency of the histone H3K36 methyltransferase SETD2 inhibits the proliferation and migration of hepatocellular carcinoma cells
Source: J Cancer. 2024 Oct 21;15(20):6479–89. doi: 10.7150/jca.97844 (PMC11632984; doi:10.7150/jca.97844)
Supplement: Supplementary file 1 — Supplementary figures. [file jcav15p6479s1.pdf]

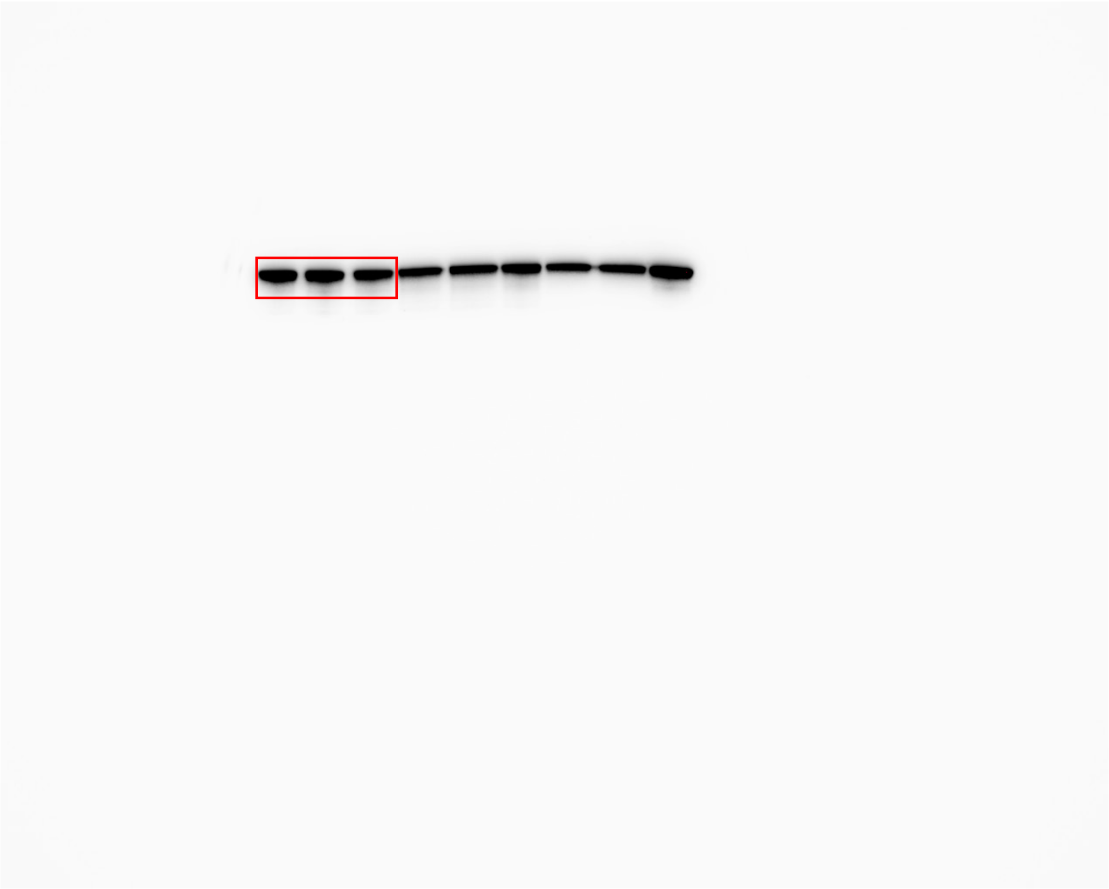

Fig. S1 original image of GAPDH western blot in figure 1B

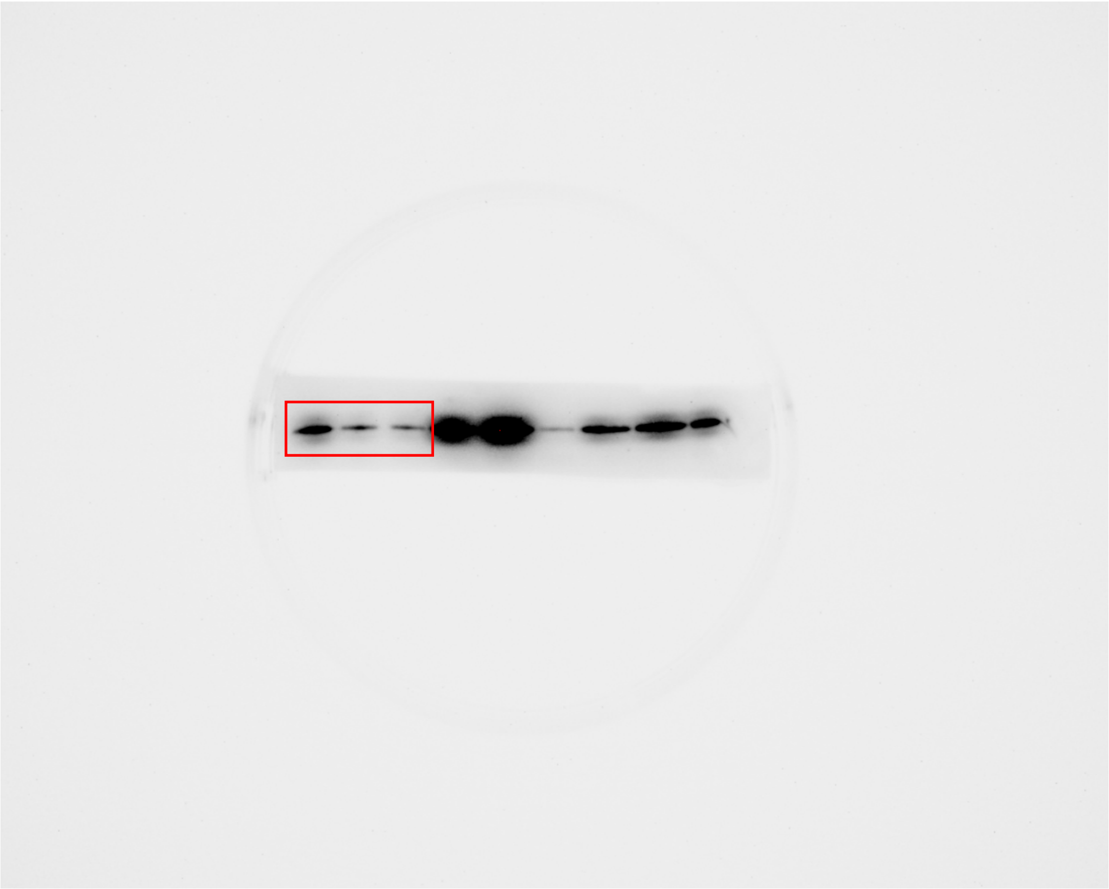

Fig. S2 original image of H3K36me3 western blot in figure 1B

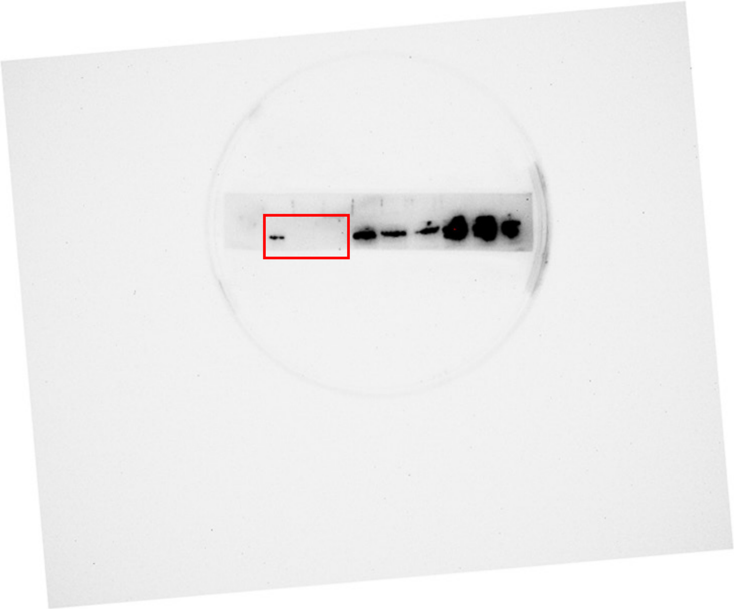

Fig. S3 original image of SETD2 western blot in figure 1B

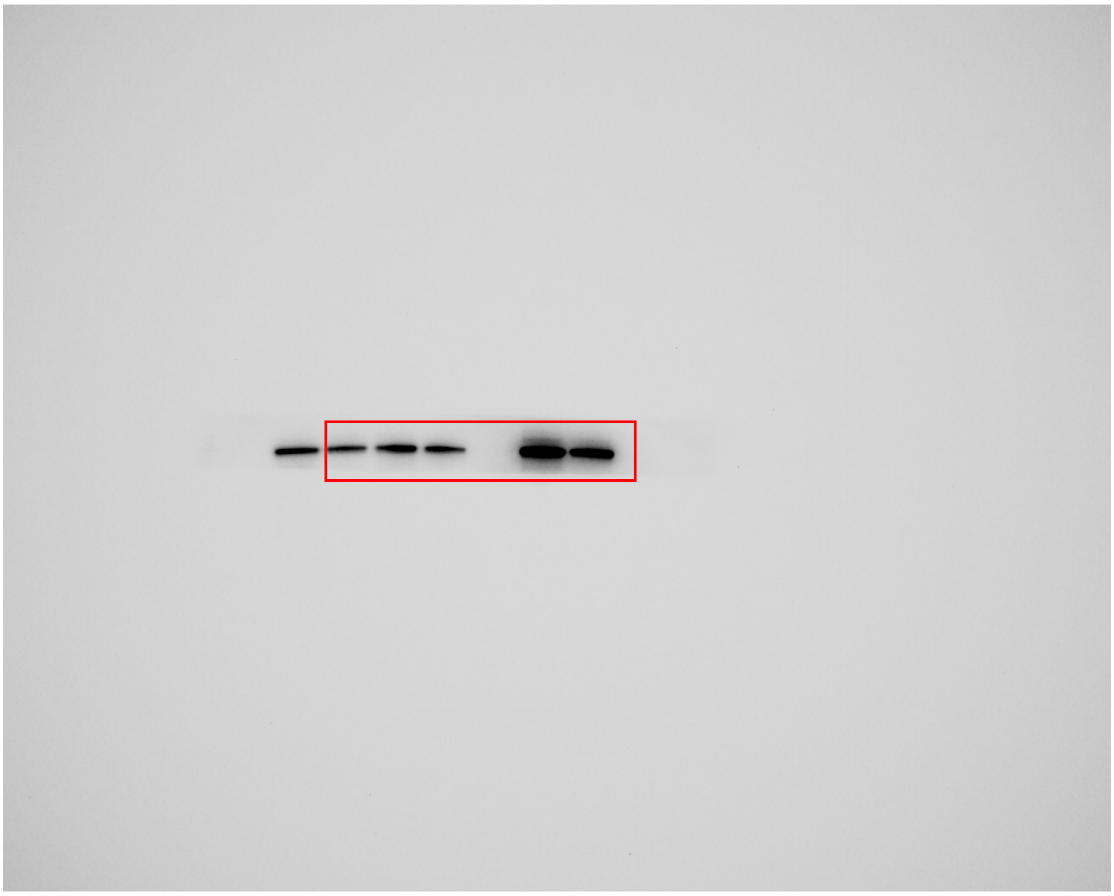

Fig. S4 original image of GAPDH western blot in figure 4I

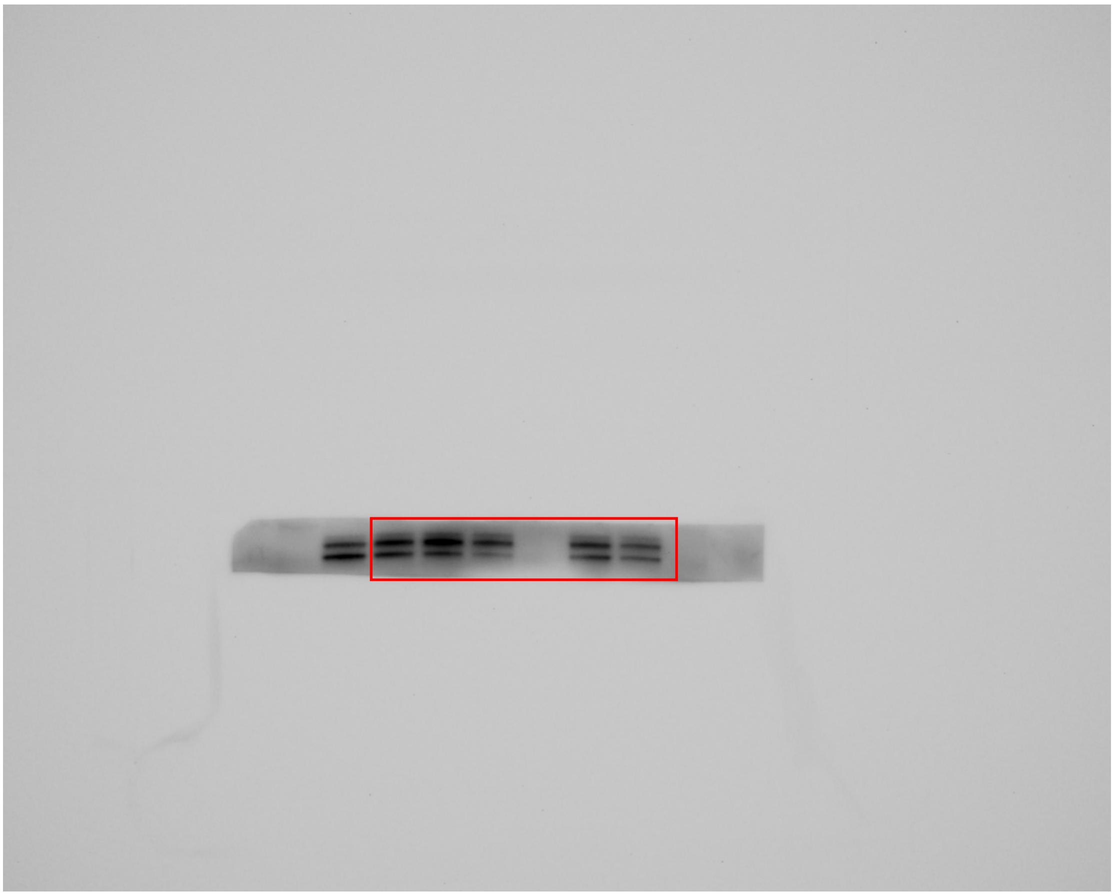

Fig. S5 original image of pERK western blot in figure 4I

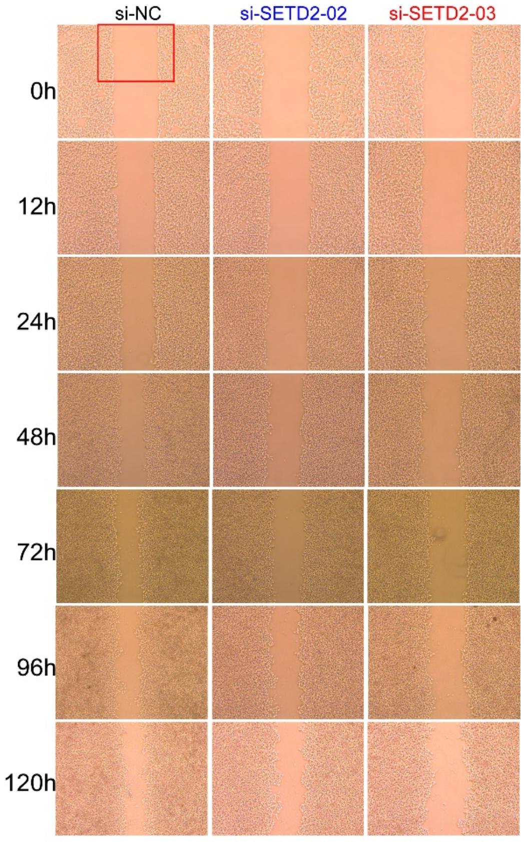

Fig. S6 original image of scratch wound healing in Figure 2E

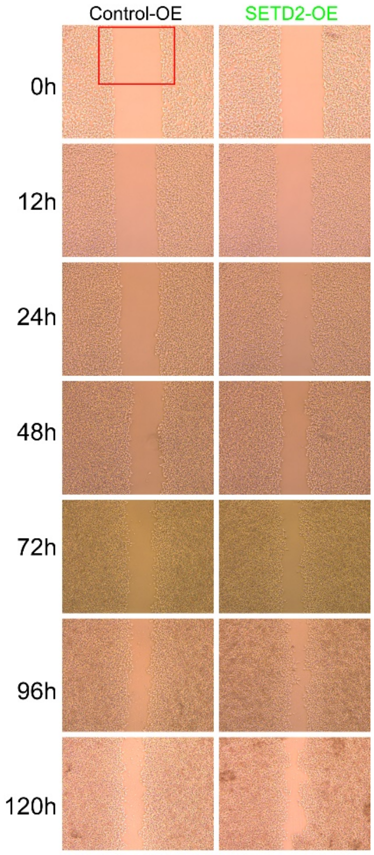

Fig. S7 original image of scratch wound healing in Figure 3D
